# A case of severe flare reaction observed in HLA B27 associated acute anterior uveitis

**DOI:** 10.1186/s12886-020-01472-3

**Published:** 2020-05-24

**Authors:** Jae-Ik Kim, Choul Yong Park

**Affiliations:** 1 Department of Ophthalmology, Dongguk University, Ilsan Hospital, 814, Siksadong, Ilsan-dong-gu, Goyang, Gyunggido 410-773 South Korea; 2 Sensory Organ Research Center, Dongguk University, Goyang, South Korea

**Keywords:** Flare, HLA-B27, Acute uveitis, AAU, Immune

## Abstract

**Background:**

Anterior chamber flare reaction refers to the light reflection from the protein in aqueous humor. We report a case of very severe flare reaction observed in human leukocyte antigen (HLA)- B27 associated acute anterior uveitis (AAU).

**Case presentation:**

An age 43 male patient visited the uveitis clinic complaining of decreased visual acuity in the right eye which developed 1 week before. The detailed ophthalmic examination revealed very severe flare reaction in the anterior chamber with diffuse conjunctival hyperemia in the right eye. Pupil margin and iris details were barely observable. Oral prednisolone 20 mg daily with topical 1% prednisolone acetate (Pred Forte, Allergan, CA) every 2 h and 1% topical cyclopentolate (Cyclogyl, Alcon, TX) three times daily were immediately prescribed. The next day, the flare reaction of the right eye decreased significantly and inflammatory cells in the anterior chamber were visible. Detailed fundus examination revealed no inflammatory signs on the retina and ciliary body. Later, the blood test revealed positive HLA B27 and autoantibodies against lupus anticoagulant with mild elevation of C reactive protein. There were no signs for ankylosing spondylitis. Continued treatment and tapering of topical 1% prednisolone acetate for 4 weeks led to the complete resolution of the anterior uveitis.

**Conclusions:**

We experienced HLA-B27 AAU with the feature of a very severe flare reaction. Conventional uveitis treatment was successful to acquire the complete resolution of the inflammation.

## Background

Anterior chamber flare reaction refers to the light reflection from the protein in aqueous humor [1]. In the inflammatory situation, the breakdown of blood ocular barrier results in increased vascular permeability of the iris and ciliary body vessels and leakage of fibrin, cytokines and other proteins [2]. Flare reaction is easily detectable using a slit lamp biomicroscopy. And laser flare photometry enables an objective quantification of the flare reaction [3, 4]. Measurement of the flare reaction is valuable for the initial diagnosis and monitoring of treatment of intraocular inflammation including various types of uveitis [2, 4–8].

In this case report, we describe a very severe flare reaction observed in human leukocyte antigen (HLA)- B27 associated acute anterior uveitis (AAU).

## Case presentation

**Fig. 1:**
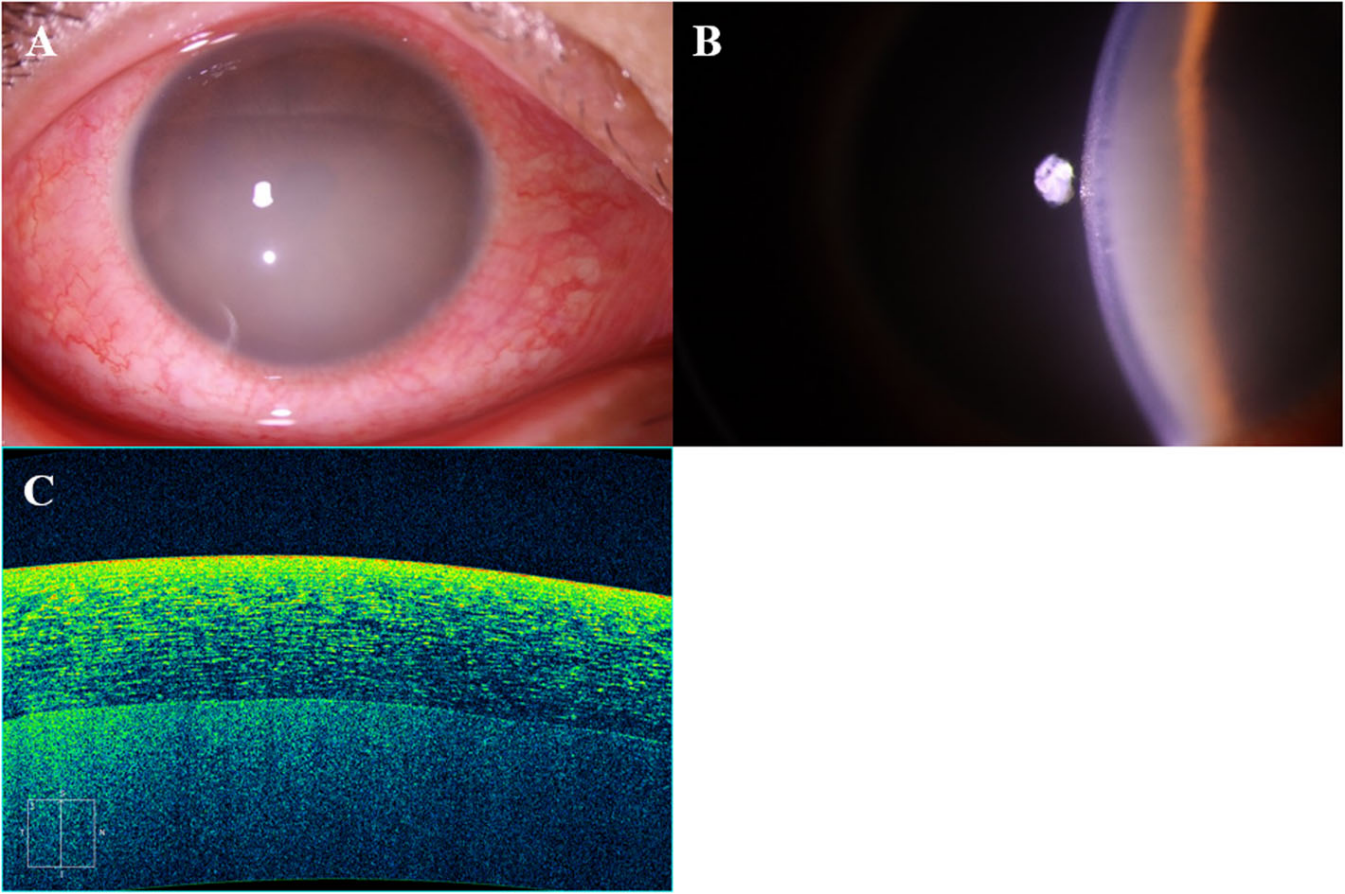
**a** Very severe flare (milky appearance) in the anterior chamber of the right eye was observed with slit lamp biomicroscopy. And pupil margin and iris details were barely observable. There was also moderate hyperemia in the conjunctiva. **b** Inflammatory cells in the anterior chamber were hardly countable because of severe haziness. **c** Severe haziness in the anterior chamber because of flare was observed in optical coherence tomography. Inflammatory cells were masked by the severe flare

**Fig. 2:**
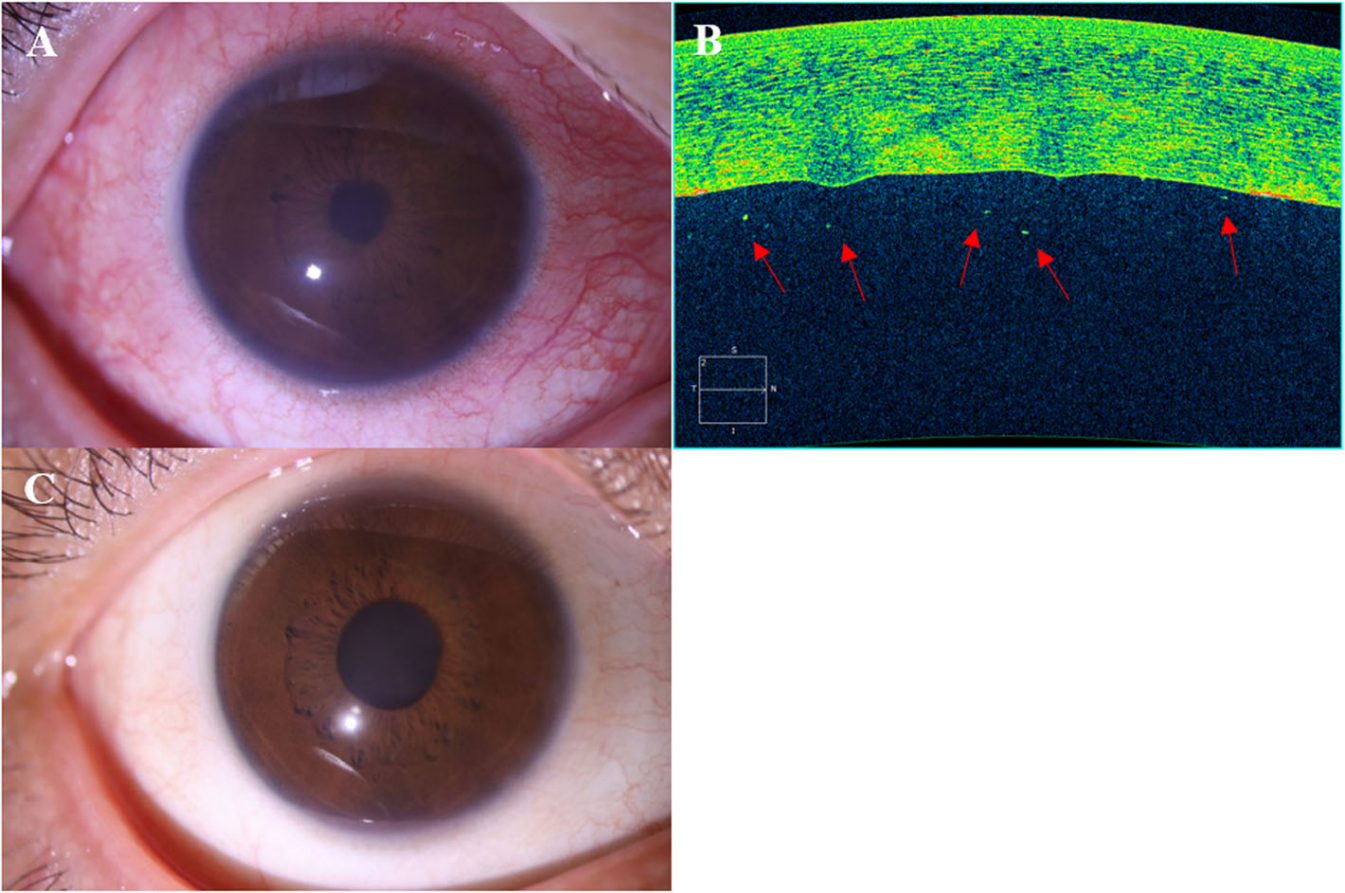
**a** After one day of treatment, the flare reaction decreased significantly in the right eye. Pupil margin and iris details were visible with mild conjunctival hyperemia. **b** Inflammatory cells (red arrows) were visible with the significant resolution of the flare in the anterior segment optical coherence tomography. **c** After 1 month of treatment, inflammation was completely resolved in the right eye

An age 43 male patient visited our uveitis clinic complaining of decreased visual acuity in the right eye. He had a history of anterior uveitis treatment of the right eye 13 years before and of the left eye 8 years before. The current episode developed 1 week before and he was prescribed topical 0.1% fluorometholone eyedrops, four times daily from the local eye clinic. On the day of the visit, he experienced acute deterioration of vision of the right eye from the early morning wakening accompanied by severe conjunctival injection. The detailed ophthalmic examination revealed the best corrected visual acuity 20/60 in the right eye and 20/20 in the left eye. Severe and diffuse conjunctival hyperemia was observed in the right eye with intraocular pressure of 16 mmHg. (Fig. 1a) Very severe flare in the anterior chamber was observed with slit lamp biomicroscopy. (Fig. 1b). It was impossible to observe or count inflammatory cells in the anterior chamber because of severe haziness. (Fig. 1c). The pupil margin and iris details were barely observable. Optic disc and retinal vessels were faintly visible. Optical coherence tomographic image of the anterior segment showed diffuse and moderate increase of corneal thickness and diffuse high signals occupying anterior chamber. He started oral prednisolone 20 mg daily with topical 1% prednisolone acetate (Pred Forte, Allergan, CA) every 2 h and 1% topical cyclopentolate (Cyclogyl, Alcon, TX) three times daily. Systemic work-up was performed including blood and urine samplings and chest X-ray to exclude etiologies including autoimmune and non-autoimmune diseases. The next day, the flare reaction of the right eye decreased significantly and inflammatory cells in the anterior chamber were visible. (Fig. 2a&b). Detailed fundus examination revealed no inflammatory signs on the retina and ciliary body. Later, blood test revealed positive HLA B27 and autoantibodies against lupus anticoagulant with mild elevation of C reactive protein. There were no signs for ankylosing spondylitis. Continued treatment and tapering of topical 1% prednisolone acetate for 4 weeks led to the complete resolution of the anterior uveitis. (Fig. 2c).

## Discussion and conclusions

According to the clinical features and blood test, the current case was diagnosed as HLA-B27 associated AAU. The prevalence of HLA-B27 varies widely between ethnic population and was reported as 7–8% in Caucasian, 4% in African, 2–9% in Chinese and 14–16% in Northern Scandinavian [9]. Frequency of HLA-B27 in Korean population was reported as 4.6–6.3% [10]. HLA-B27 is strongly associated with anterior uveitis and ankylosing spondylitis. The prevalence of HLA-B27 in AAU and in ankylosing spondylitis is thought to be up to 50 and 90%, respectively [9]. In addition, reactive arthritis, psoriatic arthritis and inflammatory bowel diseases are also associated with HLA- B27 and these conditions should also be considered as differential diagnosis in this case [9]. Herpetic anterior uveitis can be another differential diagnosis, although iris atrophy, posterior synechiae and elevated intraocular pressure are more common clinical features associated with Herpesviridae caused anterior uveitis [11].

The exact mechanism why HLA- B27 predisposes various immunologic diseases has not been fully elucidated. However, one hypothesis is that HLA-B27 encodes a number of alleles with polymorphic differences that primarily change amino acids in the antigen-binding cleft and induce a different antigen presentation which can trigger CD8 T cell proliferation [9]. Another hypothesis is that HLA-B27 has unique ability to bind some intracellular bacterial antigens such as Yersinia, Shigella, Salmonella and Chlamydia and can trigger autoimmune response [12].

The clinical characteristics of HLA-B27 associated AAU include mainly unilateral involvement, male preponderance, younger age of onset and severe fibrin reaction [13, 14]. A recent literature-based meta-analysis reported that inflammation signs such as fibrinous reaction and hypopyon were more severe in HLA-B27 positive AAU compared to HLA-B27 negative AAU [13]. Among HLA-B27 positive AAU patients, the comorbidity of ankylosing spondylitis was associated with a higher percentage of fibrinous exudation, synechiae and secondary glaucoma as compared with ankylosing spondylitis negative cases [15]. The recurrence of HLA-B27 associated AAU is high with the average frequency of annual recurrence approximately 0.8–1.1% [14, 16].

The prompt initiation of systemic steroid resulted in the rapid resolution of the flare reaction with visual acuity recovery in our case. Herbort et al. [8] reported that patients with HLA-B27-associated AAU had marked elevations of flare with the sudden onset of disease and that flare decreased rapidly to normal levels with treatment. The flare was more severe in our case, but the course of the disease was similar. However, it is noteworthy that the treatment with both topical and systemic steroids in the management of a unilateral acute anterior uveitis is controversial because it can aggravate infection-related uveitis. Therefore, careful decision making is essential. Peribulbar or subtenon steroid injection can also be useful alternative treatments [17].

The prognosis of HLA B27-associated uveitis is usually favorable. But, the final visual acuity was worse in HLA-B27 positive AAU than in HLA-B27 negative AAU. And studies have reported up to 11% of patients becoming legally blind in the affected eyes [18].

In summary, we experienced HLA-B27 AAU with the feature of a very severe flare reaction. Although the flare reaction was extraordinary, conventional uveitis treatment was successful to acquire the complete resolution of the inflammation.

## Supplementary information

**Additional file 1 **: **Supplement Figure 1**. Slit-lamp image of the right eye at 1 month after the treatment. Iris detail is clearly observed and mild cataract is visible. The anterior chamber is clear without any significant inflammatory cells.

## Data Availability

All data are available upon request to the corresponding author at oph0112@gmail.com.

## Abbreviations

AAU: Acute anterior uveitis
HLA: Human leukocyte antigen
