# Supplementary figures and images for: A case of severe flare reaction observed in HLA B27 associated acute anterior uveitis
Source: BMC Ophthalmol. 2020 May 24;20:201. doi: 10.1186/s12886-020-01472-3 (PMC7247201; doi:10.1186/s12886-020-01472-3)

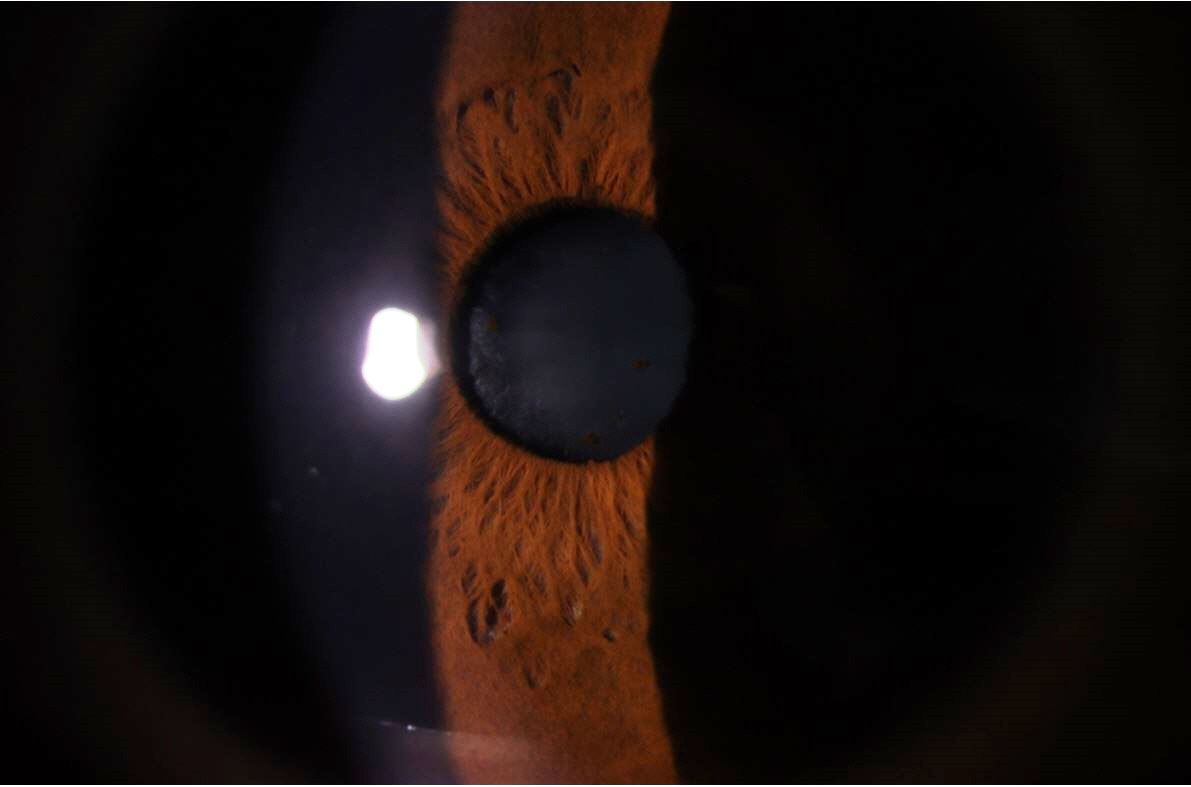

Supplement: Supplementary file 1 — Additional file 1 : Supplement Figure 1. Slit-lamp image of the right eye at 1 month after the treatment. Iris detail is clearly observed and mild cataract is visible. The anterior chamber is clear without any significant inflammatory cells. [file 12886_2020_1472_MOESM1_ESM.jpg]
